# Characterization of gait variability in multiple system atrophy and Parkinson’s disease

**DOI:** 10.1007/s00415-020-10355-y

**Published:** 2020-12-31

**Authors:** Victoria Sidoroff, Cecilia Raccagni, Christine Kaindlstorfer, Sabine Eschlboeck, Alessandra Fanciulli, Roberta Granata, Björn Eskofier, Klaus Seppi, Werner Poewe, Johann Willeit, Stefan Kiechl, Philipp Mahlknecht, Heike Stockner, Kathrin Marini, Oliver Schorr, Gregorio Rungger, Jochen Klucken, Gregor Wenning, Heiko Gaßner

**Affiliations:** 1 Department of Neurology, Medical University of Innsbruck, Innsbruck, Austria; 2 Machine Learning and Data Analytics Lab, Friedrich-Alexander University Erlangen-Nürnberg (FAU), Erlangen, Germany; 3 Department of Neurology, Hospital of Bruneck, Bruneck, Italy; 4 Department of Molecular Neurology, Universitätsklinikum Erlangen, Friedrich-Alexander University, Erlangen-Nürnberg (FAU), Schwabachanlage 6, 91054 Erlangen, Germany; 5 AG Digital Health Pathways, Fraunhofer Institute for Integrated Circuits, Erlangen, Germany; 6 Department of Neurology, Regional General Hospital Bolzano, Lorenz Boehler Street 5, 39100 Bolzano, Italia

**Keywords:** Parkinson’s disease, Multiple system atrophy, Gait variability, Gait analysis, Wearable sensors

## Abstract

**Background:**

Gait impairment is a pivotal feature of parkinsonian syndromes and increased gait variability is associated with postural instability and a higher risk of falls.

**Objectives:**

We compared gait variability at different walking velocities between and within groups of patients with Parkinson-variant multiple system atrophy, idiopathic Parkinson’s disease, and a control group of older adults.

**Methods:**

Gait metrics were recorded in 11 multiple system atrophy, 12 Parkinson’s disease patients, and 18 controls using sensor-based gait analysis. Gait variability was analyzed for stride, swing and stance time, stride length and gait velocity. Values were compared between and within the groups at self-paced comfortable, fast and slow walking speed.

**Results:**

Multiple system atrophy patients displayed higher gait variability except for stride time at all velocities compared with controls, while Parkinson’s patients did not. Compared with Parkinson’s disease, multiple system atrophy patients displayed higher variability of swing time, stride length and gait velocity at comfortable speed and at slow speed for swing and stance time, stride length and gait velocity (all *P* < 0.05). Stride time variability was significantly higher in slow compared to comfortable walking in patients with multiple system atrophy (*P* = 0.014). Variability parameters significantly correlated with the postural instability/gait difficulty subscore in both disease groups. Conversely, significant correlations between variability parameters and MDS-UPDRS III score was observed only for multiple system atrophy patients.

**Conclusion:**

This analysis suggests that gait variability parameters reflect the major axial impairment and postural instability displayed by multiple system atrophy patients compared with Parkinson’s disease patients and controls.

**Supplementary Information:**

The online version contains supplementary material available at 10.1007/s00415-020-10355-y.

## Introduction

Gait impairment, reduced mobility and falls are axial motor complications of parkinsonian disorders, more pronounced and less responsive to treatment in patients with Parkinson variant multiple system atrophy (MSA-P) than in patients with idiopathic Parkinson’s disease (PD) [1, 2]. Clinimetric rating scales such as the Movement Disorder Society revision of the Unified Parkinson’s Disease Rating Scale (MDS-UPDRS) or the Unified Multiple System Atrophy Rating Scale (UMSARS) show excellent construct validity in quantifying PD-related signs, nonetheless they provide very little information about gait impairment. For this reason, a prominent research effort over the last decades was centred on the development and validation of motion analysis technology. Importantly, senso-based gait analysis by means of inertial measurement units (IMUs) has proven to be a usable supplementary device with high sensitivity and specificity for assessing gait performance [3]. Patient-centred, wearable-derived parameters have the potential to trace prodromal axial signs, support differential diagnosis and monitor the responsiveness of treatment during interventional trials [1, 4].

To date, several studies have investigated the feasibility of IMUs in PD patients, and their clinical applicability has become evident [5]. The locomotor impairment of PD patients is reflected by alterations of gait velocity, cadence, stride time and stride length, showing progression throughout the disease course [6]. Another important domain of gait functioning is represented by its variability.

Gait variability (GV) is defined as the percent range of variance describing the regularity and consistency of a step cycle that is directly linked to dynamic postural control [7, 8]. Key measurements include variability of stride, swing and stance time, as well as stride length and gait velocity. A high GV reflects altered walking in terms of magnitude and dynamics mainly observed in movement disorders including PD [7] and Huntington’s disease [8]. Several studies demonstrated that a high GV results in walking instability and a higher risk of falling in the general elderly population [9] and in patients with PD [8]. An increased stance time and swing time variability—together with an abnormal average gait velocity—predict a higher risk of falls in PD patients regardless of medical treatment [10].

While several studies mainly focused on PD patients, only a few data have been reported for atypical parkinsonism including MSA-P. A quantitative analysis of gait parameters revealed a significant reduction of average gait velocity and stride length in patients with atypical parkinsonism compared to PD patients [11] and further observations also demonstrated increased GV [1]. Previous research described a disease-characteristic balance impairment in the medio-lateral axis displayed by patients with atypical parkinsonism [12], which is unveiled by an abnormal tandem gait test and by the information that patients stop riding a bicycle [13, 14]. However, detailed information about GV—as well as the impact of walking velocity on GV—in MSA-P patients is lacking. Although inertial sensors are not able to measure gait width and its variability yet, the available gait parameters might give us relevant insights into gait patterns of these patients.

In this study, we tested the hypothesis that MSA-P patients show a higher instability of walking reflected by an increased variability of spatio-temporal gait parameters at different gait velocities compared to PD patients and parkinsonism-free older adults of similar age. Secondly, we investigated whether walking at different velocities affects the stability of gait, evaluating gait parameters at self-paced comfortable, fast and slow walking speed between and within the groups.

## Patients and methods

This study is a post-hoc analysis of 23 patients (MSA-P *n* = 11, PD *n* = 12) enrolled in the outpatient clinic of the Department of Neurology at the Medical University Hospital of Innsbruck between June 2017 and March 2019 [4]. The study was approved by local ethics committees (IRB-approval–No 0365, 344/4.25 378/5.3, 20.10.2017) and all study participants gave written informed consent prior to participation according to the Declaration of Helsinki. Disease groups were matched by gender and Hoehn and Yahr stage, the overall inclusion and exclusion criteria are described elsewhere [4]. Briefly, the diagnosis of PD was confirmed by using the MDS criteria [15] and probable MSA-P by Gilman criteria [16]. A general and neurological examination was performed and the motor/non-motor impairment on medication was assessed using the MDS-UPDRS for PD and MSA-P patients [17], and the UMSARS for MSA-P patients [18]. To focus on gait specific items, postural instability and gait difficulty (PIGD) subscores, were calculated as sum of the items “arising from chair”, “gait”, “postural stability” and “posture” from MDS-UPDRS III [1, 19]. Specific balance testing including the Berg Balance Scale and the Timed Up and Go Test were performed and are reported elsewhere [4]. Patients were regularly followed over at least 24 months to reassess their clinical diagnosis, and one case was reclassified as PD during clinical follow‐up. The control group consisted of older adults assessed within an ongoing extension of the population-based Bruneck Study [20] performed at the Bruneck Hospital, Bolzano, Italy. Controls were clinically assessed according to a protocol used in previous Bruneck Study assessments including the MDS-UPDRS [21] and underwent sensor-based gait analysis as detailed below. Only controls free of parkinsonism and without clinically apparent gait impairment and postural instability—as measured by the corresponding items of MDS-UPDRS III—that were in the same age range as PD patients (65–80 years) were included in the present analysis (*n* = 18).

The gait trials for every participant consisted of three consecutive tasks performed uninterrupted on an overground 20 m distance (2 × 10 m walk), respectively at (a) self-paced comfortable, (b) fast, and (c) slow walking velocity. The walking pace was performed without using external cues to reflect the patients’ natural gait modulation and was done in the same order in every patient.

A detailed description of the used technical device is provided elsewhere [4, 22]. Briefly, instrumented gait analysis was performed using in-sole wearable sensors (Shimmer Research Ltd., Dublin, Ireland). Collected data was extracted by a pattern recognition algorithm for calculating clinically relevant spatio-temporal gait parameters including stride, swing and stance time as well as stride length and gait velocity (reported as the average of strides from left and right foot). We conducted a quality check of the collected data by manually reviewing the parameters for each patient and eliminating the potential technically-induced or not matching outliers. Each outlier deviating more than two standard deviations from the mean values was evaluated individually. From overall 3416 strides (on average 26.5 strides per patient per gait test), 9 strides (maximum two strides per patient per gait test) were excluded due to inconsistency or implausible values (0.44%). The coefficient of variation (CV) representing the variability of gait was calculated for the parameters stride time, percent swing and stance time, stride length and gait velocity. Mean values for stride length and gait velocity were calculated as well.

Statistical analysis was performed using IBM SPSS Statistics 24 (IBM Corp., Armonk NY). Due to non-normally distributed parameters as shown by the Kolmogorov–Smirnov test, descriptive statistics were compared using Kruskal–Wallis-Test for total *P*-values and Mann Whitney U test to compare between groups. The significance level was set at two-sided and adapted by Bonferroni correction for multiple testing. *P*-values < 0.007 were considered as statistically significant. For each gait parameter, a one-way ANOVA and Bonferroni post-hoc test were performed for group comparison. Spearman correlation analysis was conducted between gait parameters and MDS-UPDRS III as well as PIGD subscores. For within-group analysis, a one-way ANOVA for repeated measures followed by Bonferroni post-hoc test was performed.

## Results

### Patient and participant’s characteristics

The demographic data and clinical characteristics of the participants are presented in Table 1.

**Table 1:** Demographics and clinical characteristics in the disease groups

	MSA-P (*n* = 11)	PD (*n* = 12)	CG (*n* = 18)	*P *value	*P *value MSA-P vs. PD	*P *value MSA-P vs. CG	*P *value PD vs. CG
Gender (m:f)	5:6	6:6	13:5	0.292	0.880	0.238	0.325
Age	56.0 (55; 59)	74.5 (63.75; 77)	75.5 (74.75; 78)	** < 0.001**	** < 0.001**	** < 0.001**	0.134
Falls last 12 months (N)	3 (1; 10)	1 (0; 1)	0	** < 0.001**	**0.009**	** < 0.001**	**0.013**
Disease duration (years)	4 (3; 6)	7.5 (3.5; 11.5)	−	** < 0.001**	**0.037**	**− **	**− **
MDS-UPDRS II	22 (14.5; 28.25)	7 (3.25; 9)	1 (0; 2)	** < 0.001**	** < 0.001**	** < 0.001**	** < 0.001**
MDS-UPDRS III	36 (20; 62)	21.5 (16.25; 34)	5 (1.75; 7.25)	** < 0.001**	0.059	** < 0.001**	** < 0.001**
MDS-UPDRS total score	75 (47.25; 83.75)	38.5 (26.25; 49.75)	11 (7.5; 15.25)	** < 0.001**	**0.002**	** < 0.001**	** < 0.001**
PIGD subscore	4 (1.5; 6)	6.5 (3; 10.25)	0	** < 0.001**	0.069	** < 0.001**	** < 0.001**
UMSARS II	22 (14; 33)	−	**− **	**− **	**− **	**− **	**− **
Hoehn and Yahr stage	3	2.5 (2; 3)	−	** < 0.001**	0.190	**− **	**− **
LED (mg/day)	0 (0; 400)	600 (325; 858.75)	−	** < 0.001**	**0.044**	**− **	**− **

Kruskal–Wallis-Test, significance level *P* < 0.05; All values are specified as median and interquartile range (25th and 75th percentiles). Bold numbers indicate significance

*MSA-P* Parkinsonian variant of Multiple system atrophy, *PD* Parkinson’s disease, *CG* control group, *MDS-UPDRS* MDS-sponsored revision of the Unified Parkinson’s Disease Rating Scale, *PIGD* postural instability and gait difficulty, *UMSARS* Unified Multiple System Atrophy Rating Scale, *LED* Levodopa equivalent dose

All participants were able to perform the gait tasks properly without falling. The sensor-based gait analysis system automatically recognized strides for each cohort in each walking condition and stride count similarly changed with gait speed modulation within the cohorts: comfortable speed—MSA 32, PD 27, CG 25 strides, fast speed—MSA 30, PD 22, CG 19 strides, and slow speed—MSA 39, PD 27, CG 26 strides.

### Gait parameters

#### Between-group comparison

**Fig. 1:**
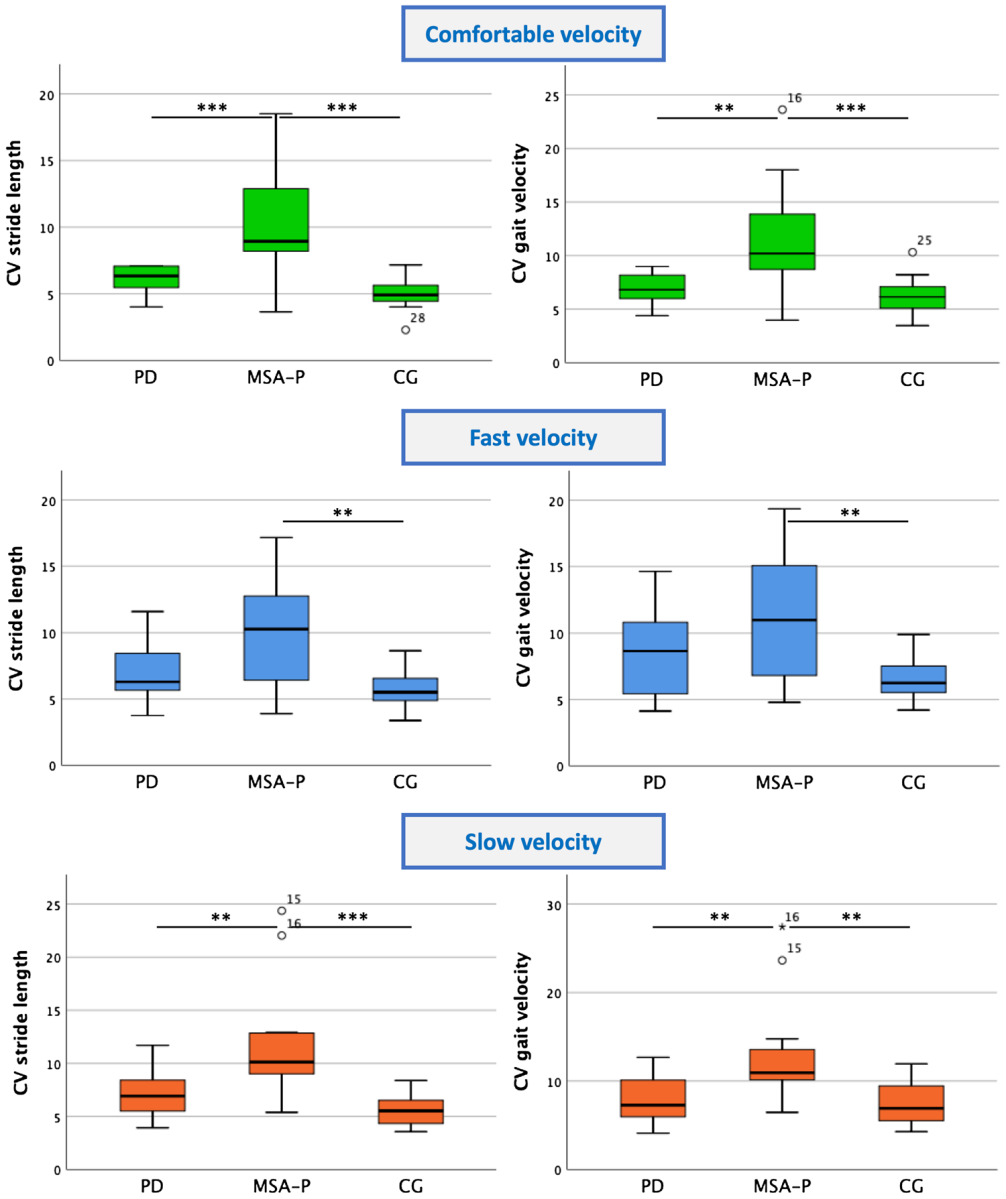
Group comparison of variability of stride length and gait velocity at different gait speeds

A detailed description of the CV parameters for the different groups is reported in Table 2. Figure 1 illustrates the comparison of the spatio-temporal parameters between the groups.

**Table 2:** Mean gait parameters and gait variability parameters at comfortable, fast and slow walking velocity

	Mean values			*P *values			
	MSA-P	PD	CG	Total	MSA-P vs. PD	MSA-P vs. CG	PD vs. CG
Comfortable speed							
Variability							
Stride time CV (%)	4.922*	3.440	3.819	0.118	0.146	0.319	1.0
Swing time CV (%)	7.546	4.520	3.929	**0.005**	0.040	**0.005**	1.0
Stance time CV (%)	3.509	2.430	2.219	0.016	0.087	0.016	1.0
Stride length CV (%)	10.588	6.101	5.089	** < 0.001**	** < 0.001**	** < 0.001**	0.827
Gait velocity CV (%)	11.668	6.896	6.207	** < 0.001**	**0.002**	** < 0.001**	1.0
Mean							
Stride length (cm)	92.61	112.09	132.94	** < 0.001**	0.069	** < 0.001**	0.021
Gait velocity (m/s)	0.792	0.999	1.199	** < 0.001**	0.109	** < 0.001**	0.074
Fast speed							
Variability							
Stride time CV (%)	4.842	4.103	4.146	0.455	0.825	0.786	1.0
Swing time CV (%)	7.541	5.291	3.813	**0.001**	0.106	**0.001**	0.350
Stance time CV (%)	4.086	3.265	2.350	**0.005**	0.427	**0.004**	0.207
Stride length CV (%)	9.911	7.004	5.569	**0.001**	0.051	**0.001**	0.527
Gait velocity CV (%)	11.285	8.333	6.631	**0.003**	0.118	**0.002**	0.527
Mean							
Stride length (cm)	104.02	130.36	150.00	** < 0.001**	0.017	** < 0.001**	0.057
Gait velocity (m/s)	1.007	1.339	1.521	** < 0.001**	0.013	** < 0.001**	0.221
Slow speed							
Variability							
Stride time CV (%)	6.005*	4.451	4.281	0.102	0.271	0.127	1.0
Swing time CV (%)	9.067	5.059	4.108	** < 0.001**	** < 0.001**	** < 0.001**	0.803
Stance time CV (%)	4.013	2.475	2.209	** < 0.001**	**0.001**	** < 0.001**	1.0
Stride length CV (%)	12.187	7.145	5.562	** < 0.001**	**0.003**	** < 0.001**	0.641
Gait velocity CV (%)	13.352	8.035	7.356	**0.001**	**0.007**	**0.001**	1.0
Mean							
Stride length (cm)	80.92	102.94	121.72	** < 0.001**	0.040	** < 0.001**	0.053
Gait velocity (m/s)	0.625	0.814	0.950	**0.001**	0.105	**0.001**	0.255

ANOVA followed by Bonferroni post-hoc test. Values in bold are marked as significant (significance level was adapted by Bonferroni correction for multiple testing, *P* < 0.007)

*CV* coefficient of variation, *MSA-P* Parkinsonian variant of Multiple system atrophy, *PD* Parkinson’s disease, *CG* control group

*Asterisks indicate within-group differences across gait speed conditions

### Comfortable speed

The mean gait velocity was significantly reduced in MSA-P patients compared to controls. No differences were found between the MSA-P and PD group. Stride length was significantly larger in the control group compared to MSA-P and PD but there was no difference between the disease groups. Regarding the CV parameters, MSA-P patients showed a significantly higher variability of swing time, gait velocity and stride length (*P* < 0.001) compared to PD patients and all values except stride time showed statistical difference between MSA-P and controls (see Fig. 1).

### Fast speed

Average gait velocity was reduced in MSA-P patients compared to PD and controls. Mean stride length was significantly higher in PDs and controls compared to MSA-P patients. As in comfortable speed, MSA-P patients showed higher CV of all parameters except stride time compared to the control group, respectively. There were no significant differences of CV parameters between PDs and MSA-Ps as well as between PDs and controls (see Fig. 1).

### Slow speed

Mean gait velocity was significantly reduced in MSA-P patients vs. controls, but not between the disease groups. Mean stride length was significantly higher in the control group than in the disease groups and in PD compared to MSA-P. As illustrated in Fig. 1, MSA-P patients showed higher values in all CV parameters except stride time compared to PD and controls. Again, no significant differences were observed for GV parameters between PD and controls.

#### Within-group analysis

**Fig. 2:**
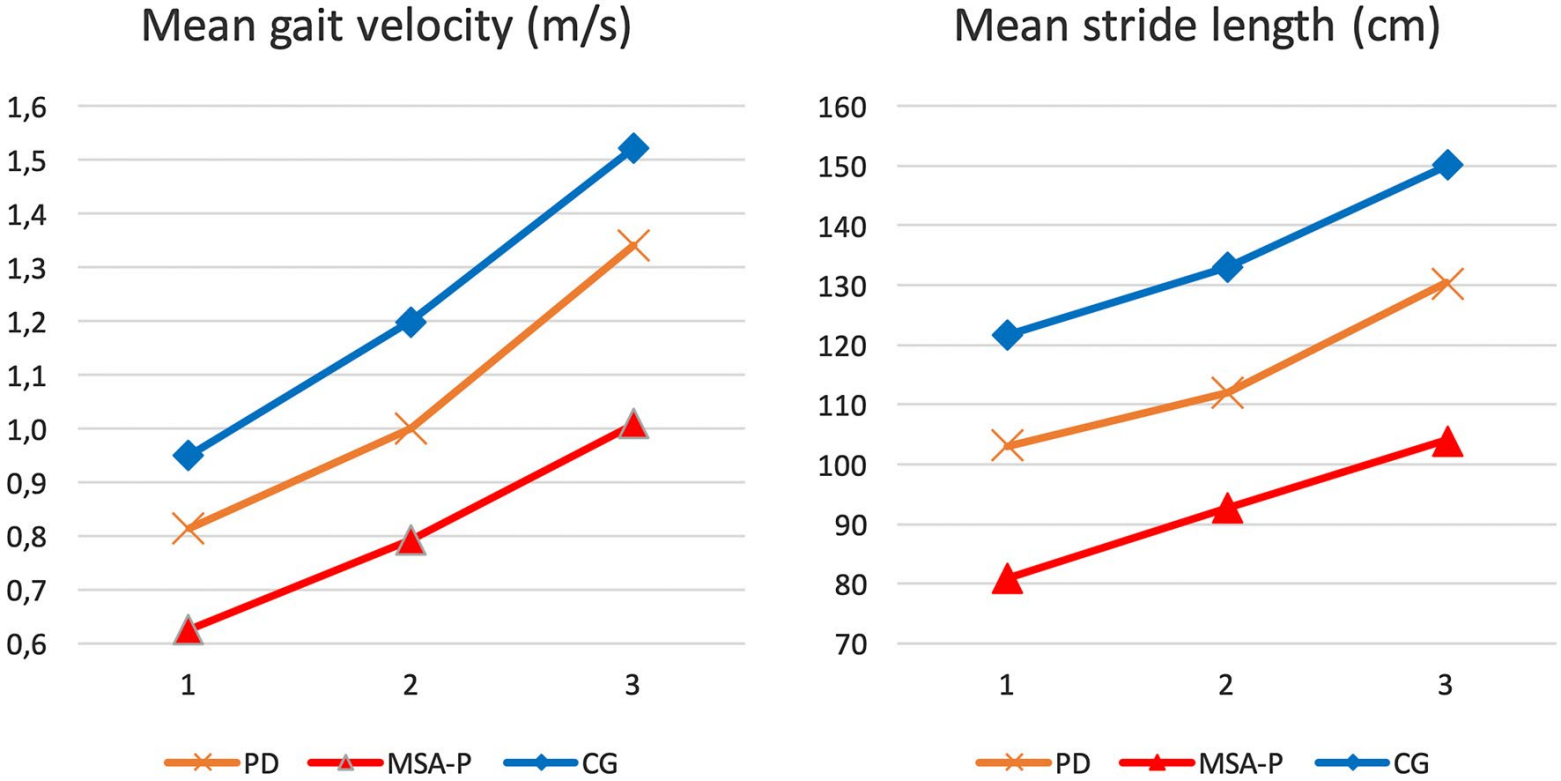
Mean gait velocity and stride length of all groups

A detailed breakdown of the modulation of mean gait velocity as well as of mean stride length are reported in Fig. 2. All study participants were able to significantly modulate their walking velocity according to the tasks (within-group differences *P* < 0.001 in every group for every task). Large inter-individual differences for gait velocity adaptations were observed (mean ranges comfortable-slow: 0.167 m/s in MSA-P, 0.185 m/s in PD, 0.249 m/s in controls; comfortable-fast: 0.214 m/s in MSA-P, 0.341 m/s in PD, 0.322 m/s in controls).

While self-paced fast gait velocity in MSA-P showed mean values of 1.01 m/s, the same velocity was used by PDs for comfortable speed (0.99 m/s) and by OAs even as slow speed (0.95 m/s).

To analyze the relative speed modulation in each group, we calculated the ratio between the velocity transitions (comfortable-to-fast = $\frac{\mathrm{fast }-\mathrm{ comfortable}}{\mathrm{comfortable}}* 100$ ; comfortable-to-slow = $\frac{\mathrm{comfortable }-\mathrm{ slow}}{\mathrm{comfortable}}*100$ ) and observed higher ranges in PD patients compared to MSA-P patients and controls (see supplementary Fig. 1).

The highest variability of gait was displayed at slow speed in MSA patients (see Table 2), where we observed a numerical increase of all CV parameters, which reached statistical significance for stride time CV in comfortable to slow speed in MSA-P patients (*P* = 0.014; effect power 0.688; see supplementary Fig. 2). Conversely, PD and controls did not show any significant differences of all CV parameters between the three gait trials.

#### Correlation of CV with MDS-UPDRS III/PIGD subscore

The correlation between gait metrics and the MDS-UPDRS III scores can be reviewed in detail in the supplementary Table 1. Correlation analysis with MDS-UPDRS III was significant for all mean and CV gait parameters at a comfortable speed in MSA-P patients, while in PD patients the scores only correlated with CV stride length. At other gait velocities, inverse correlations were observed in MSA-P patients but not in PD patients.

Correlations between gait metrics and PIGD subscores are listed in Table 3. In both MSA-P and PD patients PIGD subscores showed significant correlations with CV and mean gait parameters in comfortable and slow speed.

**Table 3:** Correlation analysis between PIGD scores and gait parameters

	PIGD-MSA		PIGD-PD	
	Rho	*P* value	Rho	*P* value
Comfortable speed				
CV Stride time	0.794	**0.006**	0.392	0.208
CV Swing time	0.754	**0.013**	0.619	**0.032**
CV Stance time	0.782	**0.008**	0.406	0.191
CV Stride length	0.609	0.061	0.641	**0.025**
CV Gait velocity	0.462	0.179	0.456	0.137
Stride length	− 0.609	0.061	− 0.740	**0.006**
Gait velocity	− 0.652	**0.041**	− 0.929	** < 0.001**
Fast speed				
CV Stride time	0.622	0.055	0.235	0.462
CV Swing time	0.732	**0.016**	0.043	0.895
CV Stance time	0.769	**0.009**	0.100	0.758
CV Stride length	0.462	0.179	0.263	0.408
CV Gait velocity	0.351	0.320	0.149	0.643
Stride length	− 0.782	**0.008**	− 0.662	**0.019**
Gait velocity	− 0.837	**0.003**	− 0.740	**0.006**
Slow speed				
CV Stride time	0.702	**0.024**	0.491	0.105
CV Swing time	0.499	0.142	0.7444	**0.006**
CV Stance time	0.665	**0.036**	0.673	**0.017**
CV Stride length	0.523	0.121	0.705	**0.010**
CV Gait velocity	0.628	0.052	0.587	**0.045**
Stride length	− 0.652	**0.041**	− 0.562	0.057
Gait velocity	− 0.726	**0.017**	− 0.760	** < 0.001**

Spearman’s correlation coefficient (rho) calculated for MSA-P and PD patients. Bold values are marked as significant

## Discussion

In this study, we tested the hypothesis that MSA-P patients display higher variability of gait at different gait velocities compared to PD patients and controls. Secondly, we investigated the within-group differences of gait metrics at three different self-paced walking velocities and correlated the clinical rating scales with the CV parameters.

One key finding of the present work is that MSA-P patients display higher GV than PD patients and controls and these differences are more distinct in slow speed, compared to comfortable or fast speed. Previous research indicated that patients with atypical parkinsonism including—but not limited to—MSA-P show significantly reduced mean gait velocity and stride length and even more pronounced differences in variability parameters compared to PD patients [1]. According to these findings, our study demonstrated that nearly all CV parameters measured in MSA-P patients were different from those obtained in PD and controls, revealing an overall greater instability of gait. The fact that mean gait velocity was not substantially different at a comfortable speed in MSA-P compared to PD while GV parameters were significantly larger in MSA-P, may indicate that GV parameters represent a more sensitive discrimination marker. Importantly, this study generates novel insights into parkinsonian gait at different walking velocities. Here, impaired balance and modulation of gait speed were demonstrated for slow and partially for comfortable but not for fast walking velocity in MSA-P compared to PD. The results of previous research exploring the influence of different walking speeds on gait variability are controversial. A number of studies in other cohorts suggested slow walking to be directly correlated with an increase in gait variability because it requires more stability in the medio-lateral axis [23]. Here, one might argue that this phenomenon may reflect the major impairment of balance affecting MSA-P patients [12]. As a consequence, tandem gait cannot be performed in the vast majority of MSA-P patients [13] and riding a bike results very difficult even in early stages [14]. However, data about the impact of fast walking are unclear. A study showed that fast walking does not increase gait variability in patients with PD, corroborating our results [24]. Although MSA-P patients are younger with theoretical age-related benefits in gait performance we observed a profound alteration of average and kinematic gait metrics that are clearly linked to the severe motor and gait impairment of the disease.

Intriguingly, while the average stride length displayed by PD patients was significantly reduced compared to controls, the same did not apply for the variability kinematics, where no significant difference was observed. These measures are apparently in contrast with previously published data, where PD show higher GV than healthy controls [8, 25]. Hausdorff et al. described an increased variability in PD patients [8] but the control group was significantly younger than the PD cohort and 60% of PD patients were in a severe disease stage (Hoehn and Yahr $\ge$ 3). Similar results were described by Rennie et al. [25], where the control group was not age matched. Our results indicate that even when gait speed changes, variability in PD and controls at the same age show similar gait stability. It is largely known that mechanical and energy expenditure optimizations change over the lifespan [26] and the effects of aging may also play a role here. That said, the impairment of average stride length displayed by PD patients may be influenced by an altered locomotor control system affecting gait stability and regularity [8, 27] or rather be a consequence of bradykinesia and lower gait speed, not intrinsic to the disease [28]. Furthermore, the fact that all our PD patients were evaluated in a stable ON phase may have positively affected gait rhythmicity, putting forth the hypothesis that dopaminergic therapy plays an important role in maintaining gait stability in PD [10].

The within-group analysis between the three walking velocities revealed an increased gait variability by reducing the gait velocity in MSA-P patients, while PD and controls showed the lowest variability at a comfortable speed. Here, it must be mentioned that all groups were able to adapt walking velocity without external cueing, as presented in significant within-group differences in gait velocity between the three conditions. Previous observations described minimal variability of gait at self-paced comfortable speed in healthy adults [29]. Indeed, this might be the walking condition with the most efficient gait and minimal metabolic energy costs. However, MSA-P patients show numerically the lowest variability in fast speed and a significant increase in CV parameters between comfortable and slow velocity. This finding supports the hypothesis that walking slowly is a more challenging task unveiling postural instability in MSA-P patients and speed modulation might not entirely be controlled by a single mechanism [8]. Potential changes in specific basal ganglia loops for integrating sensory stimuli, regulating muscle tone and performing automatic sequential movements may play a role here, but need to be further investigated.

Finally, the correlation analysis of the gait parameters with MDS-UPDRS III revealed positive moderate to strong correlations for all CV parameters in MSA-P—mainly in comfortable and slow speed, while PD patients showed hardly any correlation. Consistent with recent findings from Hasegawa et al. [30], these result may be due to the fact that the MDS-UPDRS motor score includes—beside axial—symptoms such as hypo/bradykinesia, rigidity or limb tremor, which are not closely linked with the gait metrics. Corroborating this hypothesis, a correlation analysis with the gait-focused PIGD subitems revealed significant correlations in both disease groups.

## Limitations

There are some limitations to our study. The patients’ cohorts were rather small due to the fact that MSA-P is an orphan disease and recruitment of patients who are able to walk independently without aids is challenging. Therefore, results should be interpreted with caution. However, the patients’ cohorts were well characterized and similar with regard to global motor disability. In addition, post-mortem neuropathological evaluation was not available and misdiagnosis cannot be ruled out. To minimize this problem, the clinical examination was performed by a movement disorder specialist according to the existing diagnostic criteria and patients were followed up for at least 2 years to reduce likelihood of misdiagnoses. One patient was revised from MSA-P to PD after two years, the diagnosis of all other subjects remained the same until this post-hoc analysis. MSA-P patients were significantly younger compared to the other groups. However, gait impairment in MSA-P outweighs age effects what we confirmed in a covariate analysis.

## Conclusion

To the best of our knowledge, this study was the first of its kind to evaluate gait kinematics in patients with MSA-P at different walking speed conditions. Comparing those values with PD and elderly controls free of parkinsonism and gait disorders measured by the same wearable system and with the same standardized set-ups should be acknowledged as a strength of this study, too. The present study aims to be an initial step towards gaining a more all-embracing understanding of the mechanisms that underlie gait variability in MSA-P and parkinsonism in general. Our data suggest that sensor-derived gait parameters discriminate MSA-P from PD and correlate with clinical rating scales and, therefore, provide objective research outcomes. Inertial sensors have the potential to continuously monitor gait impairment under real-life conditions of patients instead of generating snapshots in short-lasting doctoral visits. Our long-term goal is to support the visits in the hospital by continuous data of real-life scenarios to draw a holistic picture of gait impairment in everyday life. However, more research is required to determine the trade-off between validity and clinical utility of the gait variability kinematics, its dynamics and responses to interventions.

## Supplementary Information

Below is the link to the electronic supplementary material.

Supplementary file1 (DOCX 85 KB)

Supplementary file2 (TIF 1539 KB)

Supplementary file3 (TIF 1778 KB)
