# Supplementary material for: Characterization of gait variability in multiple system atrophy and Parkinson’s disease
Source: J Neurol. 2020 Dec 31;268(5):1770–9. doi: 10.1007/s00415-020-10355-y (PMC8068710; doi:10.1007/s00415-020-10355-y)
Supplement: Supplementary file 1 — Supplementary file1 (DOCX 85 KB) [file 415_2020_10355_MOESM1_ESM.docx]

Supplementary table 1: Correlation analysis between MDS-UPDRS III and gait parameters.

|  | | MSA | | PD | |
| --- | --- | --- | --- | --- | --- |
|  | | Rho | P-value | Rho | P-value |
| Comfortable speed | CV Stride time | 0.763 | **0.006** | 0.273 | 0.391 |
|  | CV Swing time | 0.671 | **0.024** | 0.336 | 0.286 |
|  | CV Stance time | 0.731 | **0.011** | 0.308 | 0.331 |
|  | CV Stride length | 0.726 | **0.011** | 0.615 | **0.033** |
|  | CV Gait velocity | 0.630 | **0.038** | 0.385 | 0.217 |
|  | Stride length | - 0.790 | **0.004** | - 0.503 | 0.095 |
|  | Gait velocity | - 0.690 | **0.019** | - 0.545 | 0.067 |
| Fast speed | CV Stride time | 0.525 | 0.097 | 0.294 | 0.354 |
|  | CV Swing time | 0.648 | **0.031** | - 0.021 | 0.948 |
|  | CV Stance time | 0.603 | 0.050 | - 0.007 | 0.983 |
|  | CV Stride length | 0.566 | 0.069 | 0.392 | 0.208 |
|  | CV Gait velocity | 0.543 | 0.084 | 0.252 | 0.430 |
|  | Stride length | - 0.872 | **<0.001** | - 0.336 | 0.286 |
|  | Gait velocity | - 0.831 | **0.002** | - 0.420 | 0.175 |
| Slow speed | CV Stride time | 0.648 | **0.031** | 0.308 | 0.331 |
|  | CV Swing time | 0.562 | 0.072 | 0.378 | 0.226 |
|  | CV Stance time | 0.489 | 0.127 | 0.406 | 0.191 |
|  | CV Stride length | 0.612 | **0.045** | 0.552 | 0.063 |
|  | CV Gait velocity | 0.708 | **0.015** | 0.343 | 0.276 |
|  | Stride length | - 0.826 | **0.002** | - 0.420 | 0.175 |
|  | Gait velocity | - 0.785 | **0.004** | - 0.573 | 0.051 |

Spearman’s correlation coefficient (rho) calculated for MSA-P and PD patients. Bold values are marked as significant.
