# Supplementary figures and images for: Characterization of gait variability in multiple system atrophy and Parkinson’s disease
Source: J Neurol. 2020 Dec 31;268(5):1770–9. doi: 10.1007/s00415-020-10355-y (PMC8068710; doi:10.1007/s00415-020-10355-y)

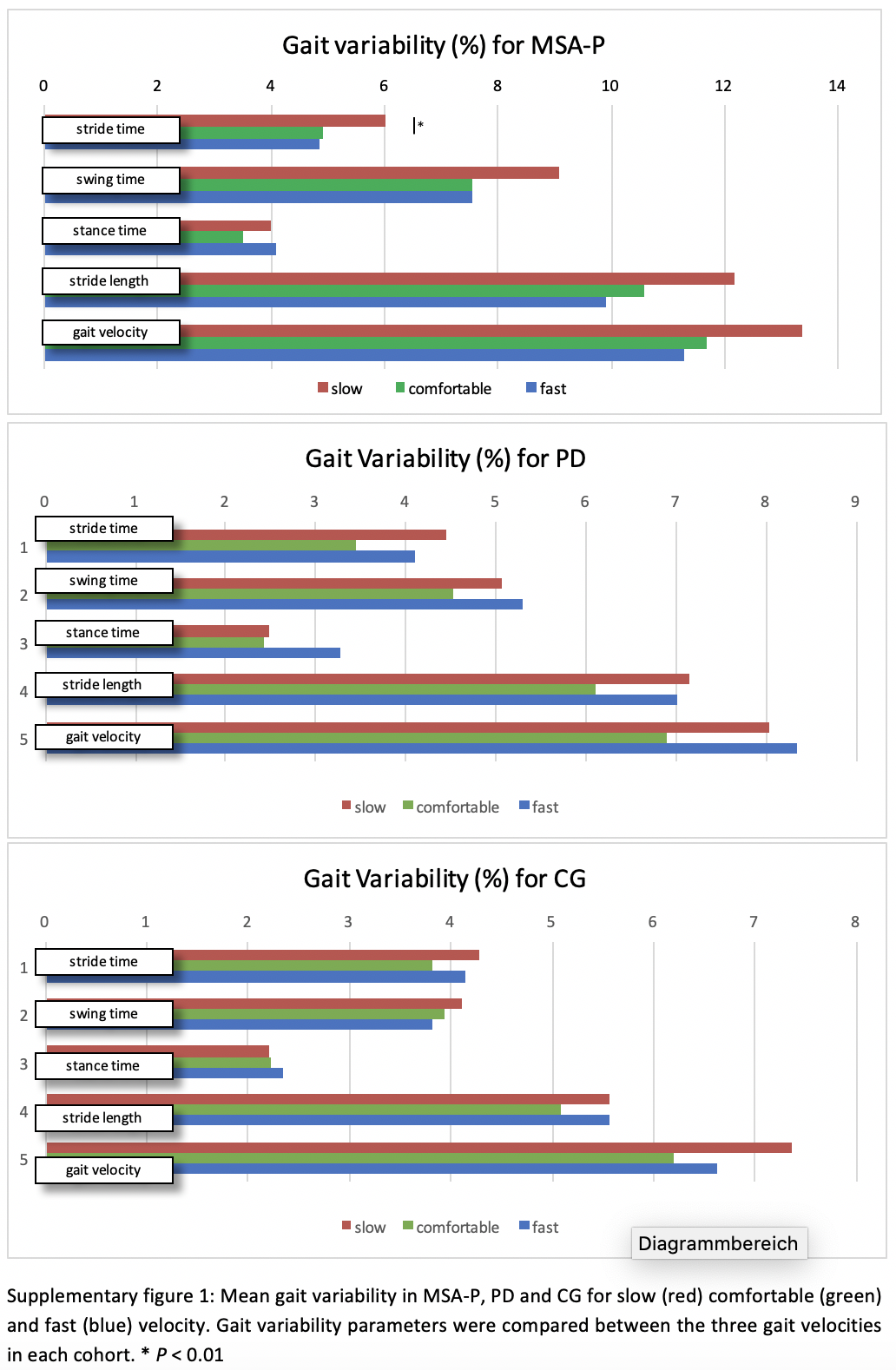

Supplement: Supplementary file 2 — Supplementary file2 (TIF 1539 KB) [file 415_2020_10355_MOESM2_ESM.tif]

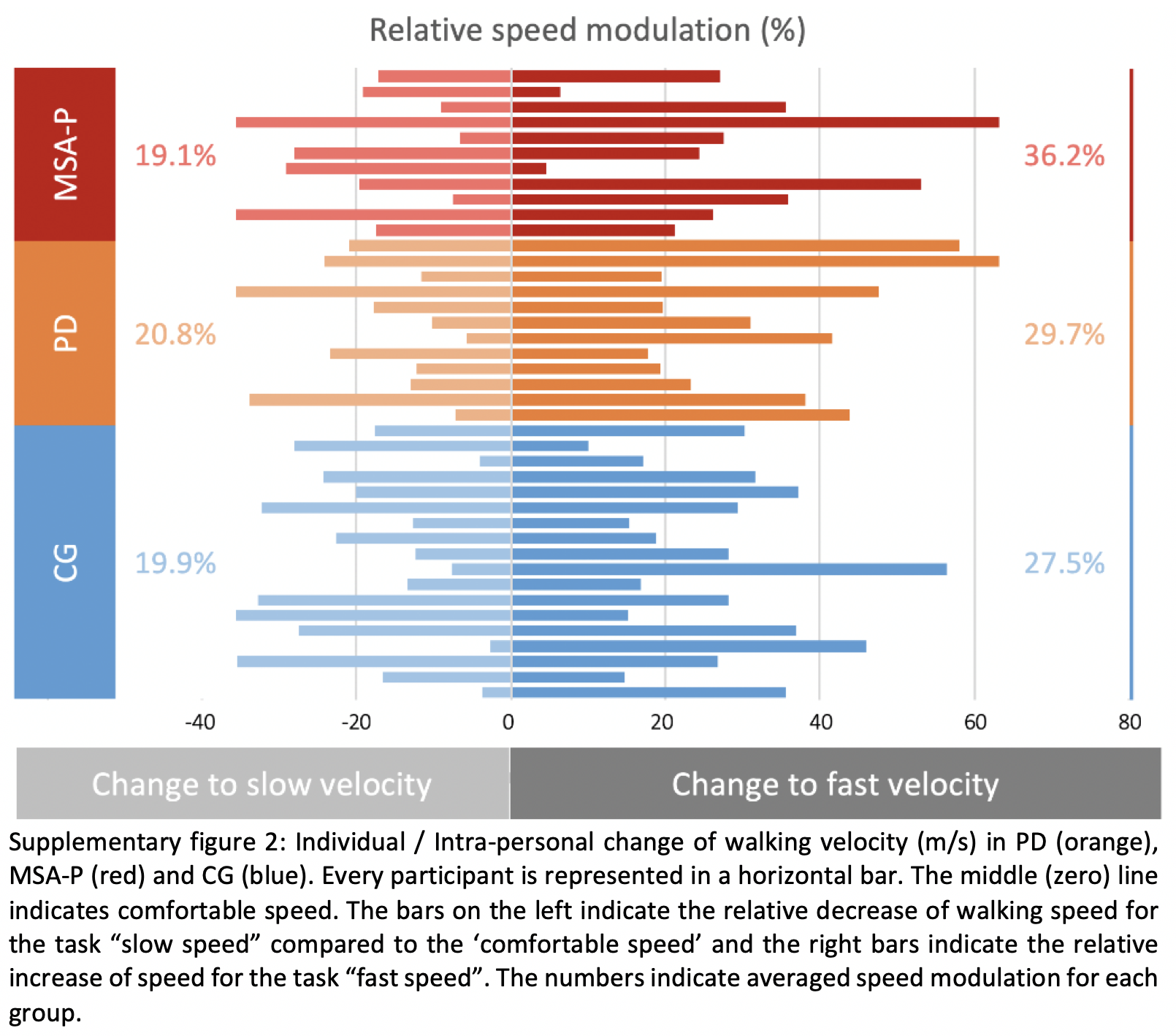

Supplement: Supplementary file 3 — Supplementary file3 (TIF 1778 KB) [file 415_2020_10355_MOESM3_ESM.tif]
